# A case of splenic artery pseudoaneurysm rupture presenting as rectal bleeding in a regional hospital

**DOI:** 10.1093/jscr/rjaa504

**Published:** 2020-12-17

**Authors:** Jonathon N Holt, Heinrich E Schwalb

**Affiliations:** General Surgery Department, Albury-Wodonga Health, Albury, NSW, Australia; General Surgery Department, Albury-Wodonga Health, Albury, NSW, Australia

## Abstract

Splenic artery pseudoaneurysm is a rare phenomenon most associated with chronic pancreatitis or previous trauma. Complications can include erosion and rupture into local structures, a situation that carries a reported mortality of 10–40%. A 58-year-old male with chronic alcoholic pancreatitis and a known splenic artery pseudoaneurysm presented to the emergency department of a regional hospital with rectal bleeding and sepsis. Computed tomography revealed a peri-splenic mass communicating with the splenic flexure. The patient was taken for an emergency splenectomy and left hemicolectomy and was confirmed to have rupture of the splenic artery aneurysm into the large bowel. This case presented with comparable features reported in the literature and demonstrates that access to emergency specialist surgical services in a regional setting offers the capability to manage rare, life threatening surgical emergencies.

## INTRODUCTION

Splenic artery pseudoaneurysm (SAP) is a rare phenomenon with an estimated prevalence amongst the general population of <1%. However, some sub-groups of patients are at much higher risk, particularly those with a history of chronic pancreatitis, pancreatic pseudocyst and previous abdominal trauma [1]. The choice of treatment for ruptured SAP has varied in the literature. We present a case herein of SAP rupture into the transverse colon presenting with rectal bleeding and sepsis in which an emergency splenectomy and left hemicolectomy were performed to control fatal haemorrhage and infection.

**Figure 1:**
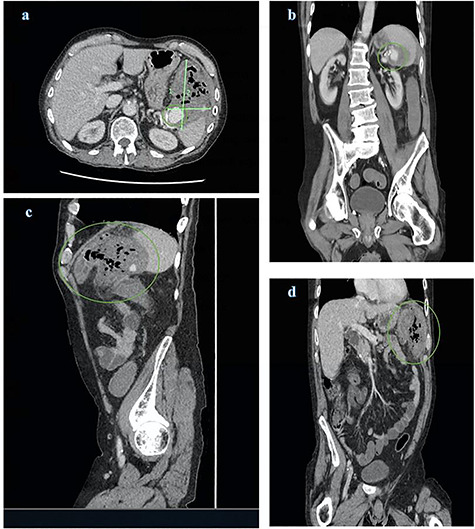
CT abdomen and pelvis views of the known splenic artery pseudoaneurysm with intravenous contrast (**a**) and (**b**) surrounded by a large inflammatory mass (a), (**c**) and (**d**); the mass demonstrates multiple foci of air and is intimately related to the splenic flexure

## CASE

A 59-year-old male presented to the emergency department of a regional hospital with a one-day history of constant epigastric pain, malaise and per-rectal bleeding. He had a known SAP on a background of multiple presentations for chronic alcoholic pancreatitis. He did not report any other symptoms. His medical conditions included chronic obstructive pulmonary disease, ischaemic heart disease and chronic hyponatraemia. The patient had been offered embolization of the SAP ~1 year prior to presentation but declined the procedure.

On examination the patient was cachectic with a bilateral course tremor of the upper limbs and had signs of septic shock with fever (39.2°C), tachycardia (127 bpm), hypotension (105/65 mmHg) and increased respiratory rate (20 bpm). The patient was tender to palpation in the epigastrium with signs of localized peritonism in the left upper quadrant. Digital rectal examination revealed plum red blood and stool.

Subsequent laboratory investigations revealed; haemoglobin, 116 g/L; white cell count, 9.4 × 10^9^/L; platelets, 277 × 10^9^/L; creatinine, 128 umol/L; international normalized ratio, 1.2; activated partial thromboplastin time, 39; and c-reactive protein, 227 mg/L.

Computed tomography (CT) of abdomen and pelvis with contrast was ordered demonstrating a large peri-splenic mass with multiple foci of air (Fig. 1). The splenic flexure appeared to communicate with the mass via the posterior wall indicative of an SAP erosion through the large bowel. The patient was taken for an emergency laparotomy with left hemicolectomy and splenectomy. The postoperative course was complicated by severe malnutrition related to chronic alcoholism and hospital acquired pneumonia resulting in sepsis unresponsive to antimicrobials. The patient requested palliation and died 3 weeks after initial presentation.

## DISCUSSION

Splenic artery aneurysms (SAA) are a pathological widening of the splenic artery, subdivided into either a true aneurysm (72%) or a pseudoaneurysm (13%) [2]. SAP differs from a true aneurysm histologically in that a collection of blood forms between the tunica media and tunica adventitia rather than a circumferential dilation of the entire vessel wall. This usually reflects an underlying process of vessel wall weakening through traumatic, inflammatory, infective or iatrogenic causes [3].

Rupture of an SAA is rare and retrospective reviews suggest that it occurs infrequently in aneurysms <2 cm in diameter [4]. Widespread use of radiological imaging has seen early detection increase, and emergent presentation with rupture has fallen from 10 to 3% [2]. Despite increasing earlier detection mortality associated with rupture remains extremely high in the range of 10–40% [5].

Complications of SAP include erosion into local structures such as the transverse colon, stomach, duodenum and pancreatic duct, with the formation of fistulous tracts causing massive gastro-intestinal haemorrhage [6]. Depending on the structures involved the clinical presentation may change significantly. In a case series review of 10 patients who presented with SAP 7/10 had bleeding, 5/10 had abdominal or flank pain and 2 patients had no symptoms at all [7]. The location of bleeding has been shown to be variable with haemorrhage noted to occur into the pancreatic duct (haemosucus pancreaticus) causing haematemesis, the lesser sac, which may present with a period of stability before circulatory collapse and the large bowel causing rectal bleeding. Less commonly patients were noted to have vomiting, nausea or a palpable mass [6].

Diagnosis is challenging and as Tessier *et al*. [4] note, many of the patients have a history of concomitant chronic alcohol abuse which may be mistakenly attributed to the presenting symptoms. Other important risk factors include previous pancreatitis, hypertension, atherosclerosis, smoking, liver cirrhosis, female sex and multiparity. CT angiography has been used extensively in diagnosis, in addition ultrasound also may be of use [7, 8]. The additional benefit of CT angiography is the ability to perform transcatheter embolization in suitable patients [6].

Numerous management strategies for ruptured SAP have been suggested including splenectomy with or without distal pancreatectomy, ligation alone and transcatheter embolization. Of these, splenectomy has been shown to have the lowest rate of failure and is likely the most durable procedure [7]. Conversely ligation alone has demonstrated the highest failure rate. In a review of visceral artery pseudoaneurysm treatment methods for 10 patients, ligation attempts ultimately failed in 6 patients (43%) [7]. Although treatment of our patient was unsuccessful due to comorbidity and post-operative infection, the treatment of the aneurysmal rupture was definitive and no re-bleed occurred.

## CONCLUSION

SAP is a rare vascular pathology most commonly associated with chronic pancreatitis. It presents clinicians with diagnostic challenges given the variability in presentation from incidental finding on imaging to unstable upper or lower gastrointestinal haemorrhage. The diagnosis should be considered in patients presenting with symptoms described above and a known history of pancreatic disease. The use of CT angiography has been established as a useful tool in aiding diagnosis of splenic artery aneurysm. The most definitive treatment has been splenectomy with few failure rates and should be considered the treatment of choice in an unstable patient indicating the importance of access to prompt surgical services, particularly in regional settings.
